# Human Serum Albumin in the Presence of AGuIX Nanoagents: Structure Stabilisation without Direct Interaction

**DOI:** 10.3390/ijms21134673

**Published:** 2020-06-30

**Authors:** Xiaomin Yang, Marta Bolsa-Ferruz, Laurent Marichal, Erika Porcel, Daniela Salado-Leza, François Lux, Olivier Tillement, Jean-Philippe Renault, Serge Pin, Frank Wien, Sandrine Lacombe

**Affiliations:** 1 Université Paris-Saclay, CNRS, Institut des Sciences Moléculaires d’Orsay, 91405 Orsay, France; xiaomin.yang@universite-paris-saclay.fr (X.Y.); marta.bolsa-ferruz@medaustron.at (M.B.-F.); erika.porcel@universite-paris-saclay.fr (E.P.); daniela.salado@conacyt.mx (D.S.-L.); 2 Université Paris-Saclay, CEA, CNRS, NIMBE, 91191 Gif-sur-Yvette, France; laurent.marichal@u-psud.fr (L.M.); jean-philippe.renault@cea.fr (J.-P.R.); serge.pin@cea.fr (S.P.); 3 Cátedras CONACyT, Universidad Autónoma de San Luis Potosí, Facultad de Ciencias Químicas, 78210 San Luis Potosí, S.L.P., Mexico; 4 Institut Lumière Matière, Université Claude Bernard Lyon 1, CNRS UMR5306, 69622 Villeurbanne, France; francois.lux@univ-lyon1.fr (F.L.); olivier.tillement@univ-lyon1.fr (O.T.); 5 Université Paris-Saclay, Synchrotron Soleil, 91190 Saint-Aubin, France

**Keywords:** nanoparticle, gadolinium, protein, structure, stability, circular dichroism

## Abstract

The gadolinium-based nanoagent named AGuIX^®^ is a unique radiosensitizer and contrast agent which improves the performance of radiotherapy and medical imaging. Currently tested in clinical trials, AGuIX^®^ is administrated to patients via intravenous injection. The presence of nanoparticles in the blood stream may induce harmful effects due to undesired interactions with blood components. Thus, there is an emerging need to understand the impact of these nanoagents when meeting blood proteins. In this work, the influence of nanoagents on the structure and stability of the most abundant blood protein, human serum albumin, is presented. Synchrotron radiation circular dichroism showed that AGuIX^®^ does not bind to the protein, even at the high ratio of 45 nanoparticles per protein at 3 mg/L. However, it increases the stability of the albumin. Isothermal thermodynamic calorimetry and fluorescence emission spectroscopy demonstrated that the effect is due to preferential hydration processes. Thus, this study confirms that intravenous injection of AGuIX^®^ presents limited risks of perturbing the blood stream. In a wider view, the methodology developed in this work may be applied to rapidly evaluate the impact and risk of other nano-products that could come into contact with the bloodstream.

## 1. Introduction

For the past decades, nanoparticles (NPs) composed of high-Z elements have been proposed to improve cancer treatments using radiation therapies [1]. Several nanoagents composed of metal oxides (iron oxide [2] and hafnium dioxide [3]), noble metals (gold [4,5] and platinum [6,7]) and lanthanides (gadolinium-based compounds [8,9]) have been developed for this purpose. 

In recent years there has been a particular emphasis placed on ultrafine NPs (< 5~10 nm) due to their enhanced tumor accumulation and renal elimination [10]. This interest was particularly illustrated by the passage to clinics of two NPs of this type: cornell dots fluorescent polysiloxane NPs, developed by Wiesner et al. [11], as an optical-PET (positron emission tomography) imaging probe for melanoma, and AGuIX^®^, a nanoagent composed of gadolinium and polysiloxane, produced by NH TherAguix (Lyon, France) [9]. The latter displays interesting properties as a contrast agent for magnetic resonance imaging (MRI) and to amplify the biological effects of high-energy photon radiation [12,13] and fast ion beams [14]. For the time being, the number of studies on this type of NP is much lower than for NPs of larger sizes.

The enhancement of radiation effects by high-Z atoms (Z_Gd_ = 64) is attributed to fast electronic processes [15,16] and consecutive localized dose deposition [12,17]. As recently reviewed [18], AGuIX^®^ opens the perspective to amplify radiation effects, as well as implement MRI-guided radiotherapy, a breakthrough in the field of cancer therapy [19]. In vivo experiments were performed to determine the maximum tolerated doses (MTD) of AGuIX^®^ in mammalians. MTDs of 450 mg/kg [20] in rodents and 750 mg/kg [21] in Cynomolgus monkeys were respectively found. During a phase Ib clinical trial (NanoRAD [9]), AGuIX^®^ was administered up to the dose of 100 mg/kg to patients, which corresponds to the recommended dose for phase 2 multicentric clinical trials (NCT03818386 and NCT02820454) [9,18] with 3 injections at 100 mg/kg at different times during radiotherapy treatment.

The enrichment of tumours with nanoagents is a critical issue. The efficiency of the intravenous (IV) injection strongly depends on the fate of the nanoagents in the body. In particular, the impact of nanoagents on metabolic routes such as enhanced permeability and retention (EPR) effect, cell uptake and renal clearance [22], strongly depends on their hydrodynamic size and surface charge [23,24,25]. When injected into patients who present blood diseases, nanoagents may cause potential harmful effects following interaction with several blood components [26]. Hence, to prevent any adverse effects that may occur during injection, the impact of the nanoagents on blood constituents is a major parameter to characterize. It has been shown that nanoagents can modify the structure of proteins and their function, increasing or decreasing their activity [27,28,29]. For instance Devineau et al. showed that ~26 nm silica NPs increase the affinity of haemoglobin for oxygen [30]. Teichroeb et al. demonstrated that the denaturation of the bovine serum albumin (BSA) is influenced by the presence of 5–100 nm gold nanospheres [31]. Laera et al. observed that the thermodynamic stability and the secondary structure of blood proteins may vary in the presence of NPs [32]. However, they found that gold nanoparticles (AuNPs) with hydrodynamic diameter ~34 nm do not impact the structure of human serum albumin (HSA) [32]. It is close to what Yin et al. also observed with ultrafine gold nanoclusters (AuNCs) [33]. On the contrary, ~15 nm silver nanoparticles (AgNPs) change the stability of HSA upon thermal treatment, modify the secondary structure of the human transthyretin (hTTR) and precipitate lysozymes [32]. Human blood proteins change conformation upon adsorption onto citrate coated AuNPs (5 to 100 nm in size) as well [34]. It is known that ultrafine AGuIX^®^ concentrates in tumors via EPR effect [22] but their impact on blood proteins is yet to be clarified. 

In this work, we present a study of AGuIX^®^ interacting with HSA. This protein is not only the most abundant in the blood plasma [35] but it also plays an important role in the regulation of the osmotic pressure and pH, as well as the transport of compounds such as fatty acids, metal ions, pharmaceuticals and metabolites. It also impacts drug delivery and detoxification [36]. The impact of AGuIX^®^ was investigated at 3100 μg/mL (47 μM), a concentration close to that expected for drug intravenous administration. The molar ratios of nanoagents to HSA (AGuIX^®^:HSA) ranged from 5 to 45 NPs per protein (5:1 to 45:1). This mimics injections of 150 to 1000 mg/kg of AGuIX^®^ corresponding to concentrations used in clinic. Dynamic light scattering (DLS) was used to determine the size of the AGuIX^®^-HSA aggregate. Synchrotron radiation circular dichroism (SRCD) was used to characterize the secondary structure and thermal stability of the protein upon addition of AGuIX^®^. The interaction of the nanoagents with HSA was characterized using isothermal titration calorimetry (ITC) and fluorescence spectroscopy. This study confirms that AGuIX^®^ does not bind to the most abundant blood protein at 37 °C, even at high concentration. The originality of this work leads to the proposal of a robust method to evaluate the safety of ultrafine nanoagents designed to be intravenously administered in clinics. 

## 2. Results and Discussions

### 2.1. DLS Measurements

**Figure 1:**
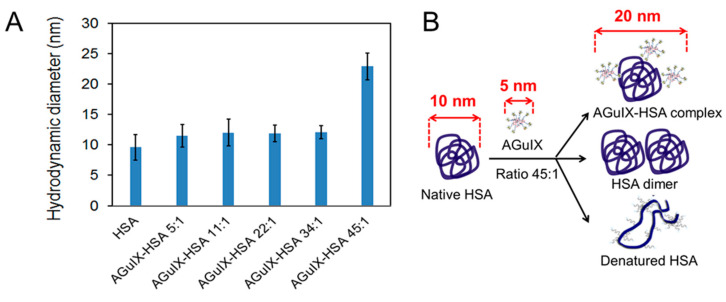
(**A**) Hydrodynamic diameters of pure HSA (human serum albumin) in the presence of AGuIX^®^ for different AGuIX^®^:HSA ratios (from 5:1 to 45:1 NPs:HSA) in 10 mM phosphate buffer at pH 7.4, T° = 37 °C. (**B**) Schematic description of the possible interactions leading to doubling the HSA diameter in the presence of AGuIX^®^ at the highest concentration (ratio of 45:1).

DLS was used to determine the hydrodynamic diameter of the objects (nanoagents, proteins, complexes) in solution and to monitor their aggregation state [37]. The diameters of pure HSA and in the presence of AGuIX^®^ at different concentrations, are presented in Figure 1A. The value for pure HSA diluted in phosphate buffer at a concentration of 3100 μg/mL was 10 ± 2 nm. This is in agreement with results reported in the literature [38]. The value of AGuIX^®^ diluted in phosphate buffer is close to 5 nm [18]. The diameter of HSA mixed with AGuIX^®^ was found similar to the value of the pure protein for ratios up to 34 NPs per protein (from ratio 5:1 to 34:1). In conclusion, AGuIX^®^ and HSA did not aggregate under these conditions. 

At the ratio of 45:1, the diameter doubled. This increase may be attributed to *(i)* the formation of AGuIX^®^-HSA complex, *(ii)* HSA dimerization or *(iii)* denaturation of HSA by AGuIX^®^ (see illustration in Figure 1B).

### 2.2. SRCD Measurements

**Figure 2:**
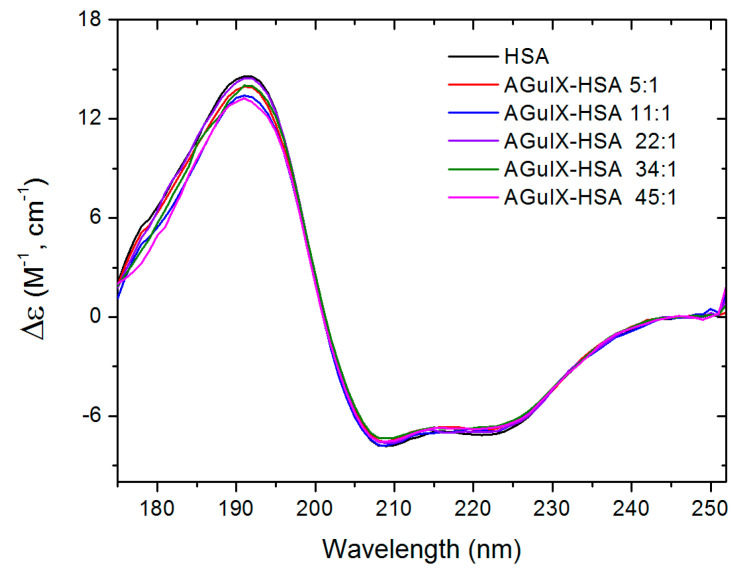
SRCD (synchrotron radiation circular dichroism) spectra of pure HSA 3100 μg/mL (black) and HSA in the presence of AGuIX^®^ at different AGuIX^®^:HSA ratios 5:1(red), 11:1 (blue), 22:1 (purple), 34:1 (green) and 45:1 (pink) in 10 mM phosphate buffer at pH 7.4 and 37 °C.

The influence of AGuIX^®^ on the secondary structures of HSA was determined by performing SRCD measurements at pH 7.4 and 37 °C. The SRCD spectra of pure HSA and HSA mixed with AGuIX^®^ at 37 °C are reported in Figure 2. The absorption of AGuIX^®^ at this wavelength range is negligible (Supplementary Figure S1).

The spectrum of pure HSA at 37 °C was found similar to the results reported in the PCDDB (Protein Circular Dichroism Data Bank) data base (Reference number PCDDB-CD 0005114000). The two negative peaks observed at 224 and 210 nm correspond respectively to the n → π* and π → π* (parallel) electronic transitions of the peptide bond [38,39]. Thanks to the wide spectral range (170–250 nm) available at the DISCO beamline–SOLEIL synchrotron (Gif/Yvette, France), a positive peak was recorded at 192 nm which is associated with the π → π* (perpendicular) transition [40]. When AGuIX^®^ was added to the solution, the peaks at 210 and 224 nm did not vary in intensity. However, a slight modification of the peak intensity was observed at 192 nm. 

The relative content of each of the four secondary protein structures (alpha, beta, turn and random coil) was determined using BeStSel analysis [41]. This tool uses the intensities of the SRCD peaks to determine the proportions of the secondary structures. The presence of three peaks (210, 224 and 192 nm) in the spectrum makes the simulation more precise than in conventional CD (circular dichroism), where two peaks are commonly reported [42]. The results obtained at pH 7.4 and temperature of 37 °C for pure HSA and HSA mixed with AGuIX^®^ at various concentrations are reported in Supplementary Table S1. 

The alpha helix content found for pure HSA at 47 μM and 37 °C was close to 53 ± 2%. This result is in agreement with the values of 67% [38], 52% [43] and 34% [44] obtained for HSA at 2 μM, 15 μM and 150 μM, respectively (a summary of the alpha helix contents as function of the protein concentration is shown in Supplementary Figure S2). In the presence of AGuIX^®^, the protein secondary structure was found constant for the ratios of AGuIX^®^ per HSA below 22:1. At higher AGuIX^®^ concentrations, the alpha helix content increased from 55% to 62% and 73% with the ratios of 34:1 and 45:1 of AGuIX^®^ per HSA, respectively. This variation suggests an interaction between the protein and AGuIX^®^ at high concentration. 

**Figure 3:**
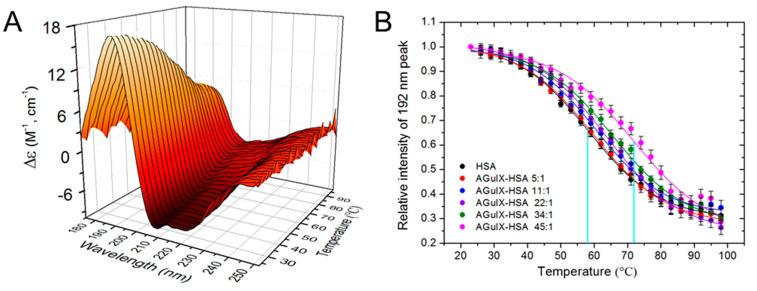
(**A**) SRCD spectra of HSA mixed with AGuIX^®^ (at a ratio of 22:1) recorded at different temperatures. (**B**) Thermal unfolding curves i.e., relative intensities of the peak at 192 nm for the pure HSA and HSA in the presence of AGuIX^®^ at different concentrations as a function of the temperature. The melting temperatures correspond to the midpoint of the respective curves (cyan vertical lines).

The melting temperature of pure HSA and HSA mixed with AGuIX^®^ was determined by recording SRCD spectra at temperatures ranging from 23 to 98 °C. The spectra obtained with the ratio 22:1 is presented in Figure 3A. Supplementary Figure S3A–E show the spectra obtained with the other ratios. The melting temperatures of the protein mixed or not with AGuIX^®^ were determined as follows. The intensities of the peaks at 192, 210 and 224 nm were collected and the values obtained at 192 nm are reported in Figure 3B (the values obtained at 210 and 224 nm are reported in Supplementary Figure S4A,B). The relative intensity curves were fitted using the Boltzmann type Equation (1) with the software OriginLab [32],

(1) $$y=A+\left(B\;-\;A\right)/\left(1+e^{\left(x-x_{0}\right)/dx}\right)$$

where *y* is the relative intensity at the temperature *x*. A and B correspond to the maximum and minimum intensities, respectively. *x*_0_ corresponds to the melting temperature of the protein and is the midpoint of the relative intensity curve. *dx* is the width of the thermal transition.

The melting temperature found for pure HSA at 47 μM (3100 µg/mL) is close to 58 °C. This value is in agreement with results reported by Das et al. for HSA at the concentration of 10 μM [45]. This value is lower than the melting temperatures of 75.1 °C and 76 °C reported by Laera et al. [32] for HSA concentrations of 0.08 μM (5 µg/mL) and 0.8 μM (50 µg/mL), respectively. It is also lower than the values of 68 °C obtained by Samanta et al. [38] for a HSA concentration of 2 μM (133 µg/mL) and 71 °C obtained by Wetzel et al. [46] for a HSA concentration of 8 μM (500 µg/mL) HSA. To our knowledge, there is no data in the literature for HSA concentrations higher than 10 μM. The decrease of the melting temperatures with increasing HSA concentrations (close to 75 °C for HSA 0.1 μM down to 55–60 °C for a concentration in the range of 10–50 μM) is in agreement with the decrease of the alpha helix content reported above (Supplementary Figure S5). It indicates that the protein gains stability by dilution.

The addition of AGuIX^®^ significantly modified the melting temperature of the protein: from 58 °C for pure HSA to 59.5 °C at the 5:1 ratio, 61.2 °C at 11:1 ratio, 63.9 °C at 22:1 ratio, 68.5 °C at 34:1 ratio and 72.7 °C at 45:1 ratio (Table 1). This variation corresponds to a gain of stability.

The influence of AGuIX^®^ on the temperature-induced variation of the HSA secondary structure was investigated. The relative contents of alpha helix, beta sheets, turns and random coils of the protein mixed with the nanoagent, as function of the temperature, were determined by performing BetSel analysis (Supplementary Figure S6). For all the samples, the alpha-helical structure became weaker as the temperature rose. The loss of 26% (from 56% at 23 °C down to 30% at 75 °C) for the pure HSA is close to the variation of 20% (from 67% at 20 °C down to 48% at 75 °C) found by Samanta et al. [38]. Interestingly, the loss of alpha helix was compensated by an increase of random coils in the temperature range of 23–58 °C, and a structuration in beta-sheets for the temperatures higher than 60 °C. Similar trends were observed in the presence of AGuIX^®^, although the structuration in beta sheet was found lower for the two highest AGuIX^®^ concentrations (34:1 and 45:1). 

**Figure 4:**
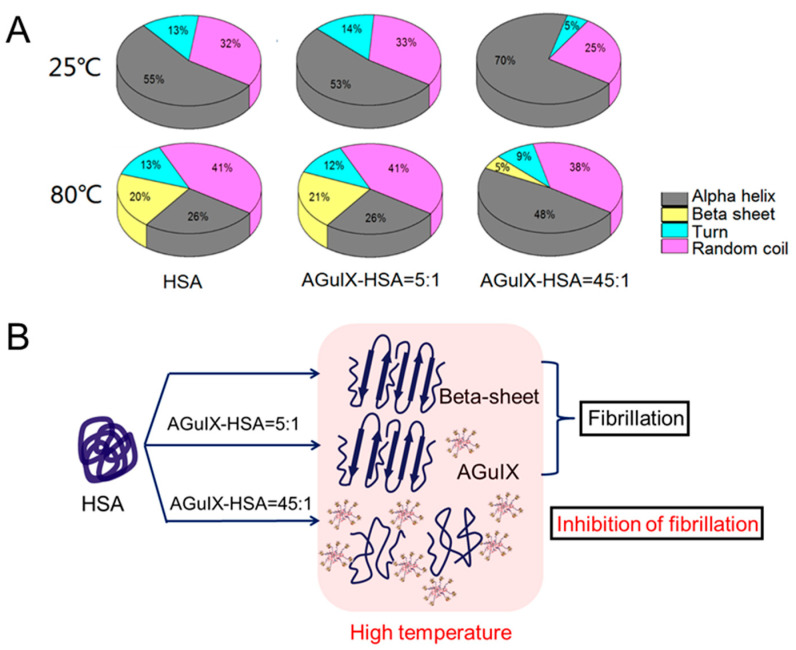
(**A**) Contribution of the respective secondary structures: alpha helix (gray), beta sheet (yellow), turn (cyan), and random coil (magenta) in pure HSA, AGuIX^®^:HSA = 5:1 and AGuIX^®^:HSA = 45:1, at 80 °C (top) and 25 °C (bottom). (**B**) Scheme of AGuIX^®^ interacting with HSA at high temperature (80 °C).

For a better understanding, a summary of the variations at two different temperatures is shown in Figure 4. 

For pure HSA, the loss of alpha helix compensated by the increase of beta-sheets from 25 to 80 °C is clearly shown (Figure 4A: yellow section). This phenomenon was already observed and is attributed to the formation of ordered beta-sheet-rich amyloid fibrils [47]. In other words, the rise of temperature favors intermolecular interaction between proteins, which rearrange to diminish the total energy of the system. Amyloid fibrils have been linked to numerous derived protein deposition neurodegenerative diseases, such as Alzheimer’s disease, Parkinson’s disease and Huntington’s disease [48]. Literature shows that NPs have both inhibitory and stimulatory activity in the process of protein fibrillation [49,50,51]. It was observed by Bag et al. [52] that graphene-based nanotherapeutics prevent the formation of HSA fibrils. However, cerium oxide NPs were found to promote protein fibrillation, as reported by Sekar et al. [53]. The addition of AGuIX^®^ at low concentration (5:1) did not modify the system and the same trend was observed as for pure HSA. At high AGuIX^®^ concentration, the fibrillation of serum albumin was limited. In this case, alpha helix content decreased less than in pure HSA and random coils replaced the loss of alpha helices. It seems that AGuIX^®^ prevents inter-protein interactions and/or stabilizes proteins until they melt.

#### Thermodynamic Analysis

The stabilization of proteins by nano-objects has been already reported in the case of polyethylene glycols, fullerol clusters and Au NPs, both in the liquid and solid phase [38,54,55]. This effect may be explained by different mechanisms [56,57,58]:

- *Option 1: enthalpy-driven mechanism*: a complex is formed between the protein and the nanoagents. In this case, the protein is stabilized due to energy release during the complex formation, which corresponds to an enthalpic stabilization. Upon heating, the protein starts unfolding after decomposition of the complex. If so, we expect an increase of the enthalpy variation of the system with increasing AGuIX^®^ concentration.
- *Option 2: entropy-driven mechanism*: the protein is stabilized due to crowding of the environment imposed by the neighbouring nanoagents, merely due to their excluded volume. This phenomenon takes place when the nanoagent hydrodynamic diameter (such as polyethylene glycol) with hydrodynamic diameter of 7.6 nm) and the protein are close [59]. In the present study, AGuIX^®^ (~5 nm), whose volume fraction ranges from 0.5 to 10%, is a possible crowder to HSA (~10 nm). Stated differently, the presence of AGuIX^®^ NPs may limit the space available for HSA to unfold and hence stabilizes the folded state by imposing an entropic penalty upon protein unfolding [60]. If so, we expect a noticeable decrease in the entropy variation of the system as a function of the AGuIX^®^ concentration.
- *Option 3: preferential hydration-driven mechanism*: as explained by Senske et al. [61], the nanoagents stabilize the native conformation of the protein relative to the unfolded state under temperature as external stress. This stabilization is attributed to an unfavourable interaction of the protein with the nanoagents, which leads to a preferential exclusion of nanoagents from the protein surface and a preferential hydration (surrounded by water molecules) of the protein. In this case, residues are more exposed to water in the unfolded state of the protein, thus the folding equilibrium is shifted toward the native state (stabilization of the protein). If so, the presence of a non-interacting agent such as AGuIX^®^ makes the unfolded HSA thermodynamically unstable, and the unfolding enthalpy variation is expected to increase with the AGuIX^®^ concentration.

In order to disentangle the different hypothesis, we determined the thermodynamic parameters, namely the enthalpy variation (ΔH°) and the entropy variation (ΔS°) of the protein, with and without NPs, using the Van’t Hoff’s equation,

(2) $$lnK=\frac{-\Delta H{}^{\circ}}{RT}+\frac{\Delta S{}^{\circ}}{R}$$

where the folding constant *K* is determined from the SRCD curves (Figure 3A) as described by Greenfield et al. [62], *T* is the absolute temperature (Kelvin) and *R* is the universal gas constant. *K* was plotted as a function of *1/T* (Supplementary Figure S7). The unfolding enthalpy variation ΔH° and the entropy variation ΔS° of the system were determined (see details in Supplementary Materials) from the slope and intercept (see Table S2) of the Van’t Hoff’s plot, respectively. The data are presented in the Table 1.

This analysis shows that ΔH° and ΔS° values increased up to 10% with AGuIX^®^ concentrations. This excludes option 2 where a decrease of the entropy variation is expected. Hence, the increase of the enthalpy variation and the entropy variation may be explained only by options 1 or 3. 

To distinguish between these two remaining mechanisms, isothermal titration calorimetry (ITC) and fluorescence spectroscopy were performed. 

### 2.3. ITC Measurements 

**Figure 5:**
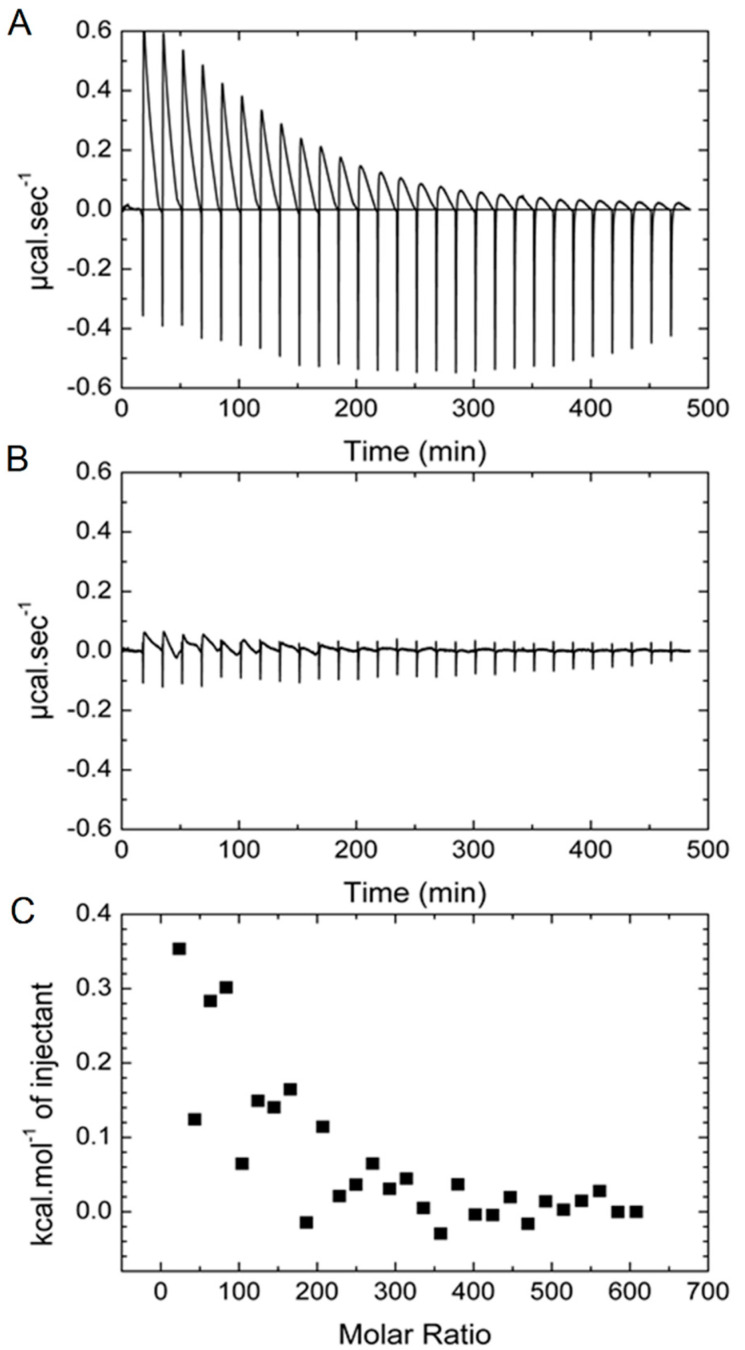
(**A**) Heat exchanges raw data of the addition of 10 µL aliquots of AGuIX^®^ (7.5 × 10^−3^ M) in a HSA solution (3 × 10^−6^ M). (**B**) Heat exchanges obtained after subtraction of the control experiments (titration of AGuIX^®^ solution to buffer, buffer into HSA, and buffer into buffer). (**C**) Titration data calculated from the subtracted data. Each experiment was done at 20 °C.

The results of the ITC experiments are displayed in Figure 5A. This figure shows the time-dependent evolution of heat upon injection of an aliquot of AGuIX^®^ into protein solution. The raw heat exchange data show highly exothermic peaks that decrease slowly (~1000 sec to reach equilibrium). Very similar peaks were found in the control experiment (injection of AGuIX^®^ in phosphate buffer). Part of the signal corresponds to the mere dilution of AGuIX^®^. However, due to its slow kinetics, the majority of the signal can be attributed to the dissolution of the AGuIX^®^ silica cores as was established by high-performance liquid chromatography (HPLC) and relaxometry experiments [63,64].

After subtraction of the control, very small exothermic peaks can be observed at the beginning of the titration (Figure 5B). The integration of the titration data as a function the AGuIX^®^:HSA molar ratio are reported in Figure 5C. Based on the fact that proteins can modify the dissolution mechanisms of silica [65], the beginning of the titration curve can be considered as a shift in the dissolution process. Therefore, this analysis demonstrates that there is not strong interaction between AGuIX^®^ and HSA.

Additional fluorescence measurements were performed to probe the formation of a complex between AGuIX^®^ and HSA.

### 2.4. Fluorescence Spectroscopy

**Figure 6:**
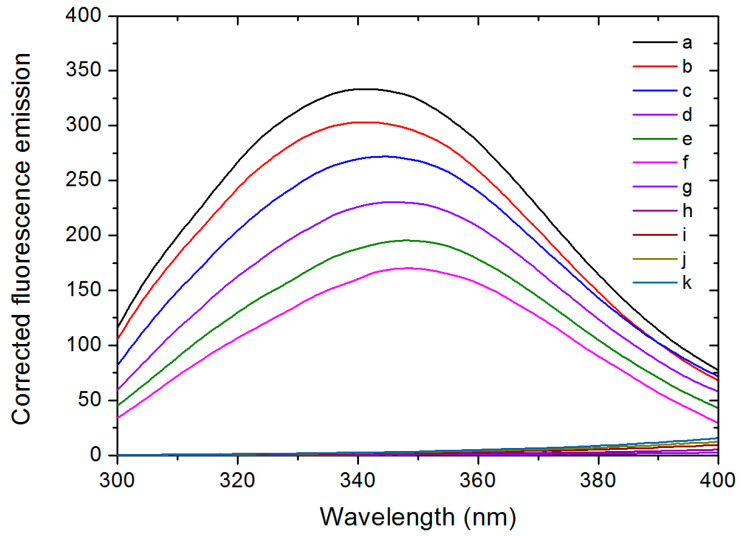
Corrected fluorescence emission spectra of HSA without (a) and with various AGuIX^®^ concentrations, AGuIX^®^:HSA ratios from 5:1 to 45:1 (b–f) and AGuIX^®^ alone at corresponding concentration (g–k) in 67 mM phosphate buffer at pH 7.4, 25 °C and upon excitation at 280 nm.

The fluorescence emission spectra of pure HSA and HSA mixed with various concentrations of AGuIX^®^ were measured at 25 °C and 37 °C. The fluorescence signal of AGuIX^®^ is negligible (Figure 6g–k). However, AGuIX^®^ absorbs in the range of 300–400 nm (see UV–vis absorption spectra of AGuIX^®^ in Supplementary Figure S8A,B). So, the fluorescence spectra were corrected by the absorption of the inner filter effect of AGuIX^®^ following the Lakowicz’s method [66]:

(3) $$F_{cor}=F_{obs}10^{\left(A_{em}+A_{ex}\right)/2}$$

where *F_cor_* and *F_obs_* are the corrected and the measured fluorescence intensities, respectively, while *A_ex_* and *A_em_* are the absorbance at the excitation (280 nm) and emission wavelengths (343 nm). The results obtained at 25 °C are shown in Figure 6 (Supplementary Figure S9 for the results at 37 °C).

The corrected spectra displayed a maximum around 340 nm, which mainly corresponds to the fluorescence of the tryptophan residue [67]. A red shift of the peak maximum (from 340 nm to 350 nm) was observed simultaneously. This suggests that the environment of the fluorescent chromophore, the tryptophan residue, became more polar/hydrophilic after addition of AGuIX^®^ [68,69]. As tryptophan residues are highly susceptible to the microenvironment, diverse molecular interaction may induce fluorescence quenching [70]. The intensity of the peak decreased gradually with increasing concentration of AGuIX^®^ (~52% decrease for the AGuIX^®^:HSA ratio of 45:1 compared to pure HSA), which highlights the quenching effect of AGuIX^®^ on the HSA fluorescence. 

**Figure 7:**
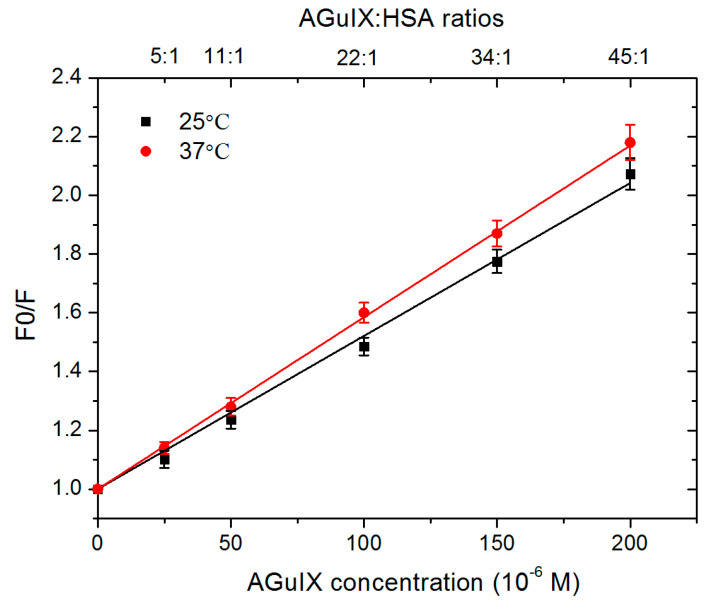
Stern–Volmer plots (F_0_/F) obtained at 25 °C (black) and 37 °C (red) as function of AGuIX^®^:HSA ratios, from 5:1 to 45:1.

The quenching of the protein fluorescence by a quencher (e.g., AGuIX^®^) may be due to two possible mechanisms: (i) the formation of a complex between the quencher and the protein (static quenching) and (ii) collisions (diffusion limited) between the quencher and the protein (dynamic quenching) [71]. These two mechanisms can be differentiated by measuring the temperature dependence of the quenching intensity [66]. In this purpose, the fluorescence data were analysed using the Stern–Volmer equation [71]:

(4) $$\frac{F_{0}}{F}=Ksv\left[Q\right]+1=kq\tau_{0}\left[Q\right]+1$$

where *F_0_* and *F* are the steady-state fluorescence intensities (at around 340 nm) of HSA in the absence and in the presence of a quencher, respectively. *K_SV_* is the Stern–Volmer constant (in M^−1^), *[Q]* is the quencher concentration (in M), *kq* is the experimental quenching rate constant (in M^−1^.s^−1^) and τ_0_ is the excited-state lifetime (in sec) of HSA in the absence of quencher. Figure 7 displays the Stern–Volmer plot of the AGuIX^®^:HSA systems obtained at two different temperatures, 25 °C and 37 °C.

The *Ksv* and, consecutively, *kq* values were determined considering τ_0_ ~ 6.38 × 10^−^^9^ s [72]. These values are reported in Table S3. 

The Stern–Volmer constant (*Ksv*) and the quenching rate constant *(kq*) increased with the temperature. This result is indicative of a dynamic quenching of HSA fluorescence by AGuIX^®^. In this case, collisions between the two entities are responsible for the increasing quenching phenomenon. In the case of a static quenching, an increase of the temperature would dissociate the complex quencher-protein. This would result in a decrease in the fluorescence quenching (*Ksv* and *kq*) and an increase in the fluorescence yield [73], which is different from our results. This result is in agreement with the dynamic quenching of HSA by AuNPs reported by Foo et al. and Yin et al. [74,75]. 

Finally, the ITC experiments together with fluorescence emission spectroscopy confirm that AGuIX^®^ do not bind to HSA (non-complex formation). The stabilization of HSA by AGuIX^®^ NPs at high concentration (45:1) may be explained by a preferential hydration process [61]. This effect has been already observed for small solutes interacting with proteins [76]. However, it is here for the first time suggested for a nanoagent. This observation is important with respect to the unwanted immune response triggered by many nanoagents. These responses, usually raised by the complement system, have been connected to their tendency to interact with proteins and favor their unfolding [77]. AGuIX^®^ paves the way to a new generation of nanoagents where this effect will be alleviated.

## 3. Conclusions

This paper focuses on the dynamic and mechanistic interaction between HSA and AGuIX^®^, a nanoagent currently tested in clinics as an MRI contrast agent and booster of radiation therapy. The influence of the AGuIX^®^ concentration was investigated using AGuIX^®^:protein ratios that correspond to those expected during patients’ intravenous injections, with the highest tested AGuIX^®^ concentration ~20 times higher than the concentration currently used in clinics. We systematically examined the interaction of HSA with AGuIX^®^ using complementary techniques. DLS measurements indicated an increase in the hydrodynamic diameter of HSA upon incubation with AGuIX^®^ at the highest concentration. This increase suggests a possible AGuIX^®^-HSA binding. Further on, ITC analysis revealed no strong interaction between AGuIX^®^ and HSA and small exothermic peaks were attributed to an AGuIX^®^ mere dilution. Fluorescence quenching investigation substantiated that AGuIX^®^ do not bind with the HSA. Fluorescence quenching of serum albumins by AGuIX^®^ was associated with a dynamic process. In addition, the thermal analysis performed with SRCD showed that there is a significant increase of the HSA melting temperature when AGuIX^®^ is added at high concentrations (34:1 and 45:1 AGuIX^®^: protein ratios). This result underpins an increased protein stability. We demonstrated that it is due to a preferential protein hydration. As NPs are increasingly prevalent in biomedical applications, a thorough understanding of their effects on the structure and functions of proteins is essential. Moreover, this experimental protocol (combination of ITC, CD and fluorescence) should be considered in the near future to evaluate the interaction of any NP with proteins. Ultimately, in line with economical and ethical issues, this method could be proposed as a test prior to in vivo experiments. 

## 4. Materials and Methods

### 4.1. AGuIX^®^ Nanoparticle 

The synthesis and main characteristics of the AGuIX^®^ have been previously reported [78,79]. AGuIX^®^ are composed of a polysiloxane network surrounded by Gd chelates similar to DOTA (1,4,7,10-tetra-azacyclododecane−1-glutaric anhydride-4,7,10-triacetic acid) that are covalently grafted to an inorganic matrix. AGuIX^®^ has a hydrodynamic diameter of 5 ± 3 nm for a mass around 10 kDa and has a slightly positive zeta potential at pH 7.2. These highly stable NPs can be lyophilized and stored at 4 °C for years. They were re-suspended in water at room temperature 24 h before their use.

### 4.2. Human Serum Albumin (HSA)

HSA was purchased from Sigma-Aldrich. The protein was re-suspended in water and dialysed against a phosphate buffer (10 mM, pH 7.4) at room temperature. The concentration of HSA was determined by absorption spectroscopy and reassessed by quantitative amino acid analysis (QAA) at Pasteur Institute (Paris, France). A concentration of 3875 μg/mL (59 μM) was reached. 

### 4.3. Preparation of the Samples

A final HSA concentration of 3100 μg/mL (47 μM) was used in all the experiments (to be compared with the 35~50 mg/mL in blood). High concentrations of AGuIX^®^ were used to mimic the initial condition of an intravenous injection. In this perspective, ratio of AGuIX^®^ to HSA of 5:1 up to 45:1were prepared.

### 4.4. Synchrotron Radiation Circular Dichroism (SRCD)

#### 4.4.1. Sample Loading

Samples of 4 µL, containing 12.4 µg of HSA each and various amounts of AGuIX^®^, were loaded in circular demountable CaF_2_ cells [80] of 23.4 µm path length. Initial static SRCD spectra were acquired at 37 °C. 

#### 4.4.2. Spectra Acquisition

The SRCD measurements were performed at the DISCO-beamline, SOLEIL Synchrotron (Saint-Aubin, France). Thanks to the photon flux and beam size at the synchrotron facility [81], the measurements were performed with a good signal to noise ratio (200) down to 175 nm, which is difficult to reach with a conventional CD set up [82]. (+)-camphor-10-sulfonic acid (CSA) was used to calibrate the amplitudes and wavelength positions to perform optimized SRCD experiments. We acquired the spectra of our samples from 250 down to 175 nm every nanometer using a 1 nm bandwidth and 1.2 s integration time. Raw data (θ) was measured in millidegrees (mdeg), which were converted to molar circular dichroism values (Δε), after averaging, baseline subtraction and calibration with CSA. For the molar circular dichroism values (Δε, M^−1^.cm^−1^ = L.mol^−1^.cm^−1^), the following equation was applied:

(5) $$\Delta \varepsilon =\;\theta \;\times \frac{\left(0.1\times \mathrm{MRW}\right)}{\left(P\times C\right)\times 3298}$$

where the MRW (mean residue weight) of HSA is 113.8 Da (g.mol^−1^), P (pathlength) is 0.00234 cm (measured by interferometry) and C (protein concentration) is ~3.1 mg/mL. Spectral acquisitions of 1 nm steps at 1200 ms/nm integration time were performed three times for each sample (including the baseline).

#### 4.4.3. Thermal Denaturation

Thermal denaturation experiments were carried out by collecting SRCD spectra at temperatures ranging from 23 to 98 °C using a 3 °C ramp and a 5 min settling time. Three spectra were collected for each temperature. Each measurement lasted for 3 h. In order to assure that over this time no additional phenomena (radiation damage or drying) affected the spectral amplitude, spectra were also recorded for 3 h without changing the temperature. This showed that the protein was not susceptible to radiation damage and that the photon flux and beam size were appropriate (~10^11^ photons/s at 2 × 2 mm^2^). Single value decomposition indicated two principal components (spectra) suggesting a two-state transition. Three separated data collections with fresh sample preparations were carried out for consistency and repeatability.

#### 4.4.4. Spectra Analysis

Data treatment including average, baseline subtraction, smoothing, scaling and standardisation were performed with the CDtool software [83]. Secondary structures content was determined using the ContinLL and SELCON3 program [83], SP175 as reference setting and additionally using the BestSel web server [42]. Normalized root-mean-square deviation (NRMSD) indicated the most accurate fit for each spectrum. Values of <0.15 were considered significant. 

Calibrated and normalized spectra were submitted to the publicly accessible PCDDB database [84] under reference CD0005114000.

### 4.5. Isothermal Titration Calorimetry (ITC)

Adsorption isotherms of HSA on AGuIX^®^ were performed by calorimetry using a MicroCal VP-ITC (GE Healthcare). Before the measurements, all the solutions were degassed under vacuum. The reaction cell (1.8 mL) was loaded with a HSA solution at 3 μM. The syringe (500 μL) was filled with an AGuIX^®^ solution at 7.5 mM. The proteins and NPs were prepared in the same phosphate buffer (pH 7.4) to prevent any pH effect. The experiments were done in duplicate at 20 °C by adding 10 μL of AGuIX^®^ solution to the HSA solution with an equilibration interval of 1000 s. The control experiments were performed without AGuIX^®^ on one hand and without HSA on the other hand. The heat of dilution measured was subtracted from the titration data. The ITC experiments were performed at 20 °C.

### 4.6. Fluorescence Quenching Studies

The interaction of AGuIX^®^ with HSA was studied using fluorescence quenching studies of HSA by AGuIX^®^ NPs. The concentration of HSA was maintained at 4.7 μM, while the AGuIX^®^ concentrations varied to reach AGuIX^®^:HSA ratios from 5:1 to 45:1. The final volume of reaction mixture was made up to 1 mL with 67 mM sodium phosphate buffer, pH 7.4. The samples were allowed to equilibrate for 30 min prior to fluorescence measurements. The fluorescence spectra of these samples were recorded at 25 °C or 37 °C on a Cary Eclipse Fluorescence Spectrofluorometer using a quartz cuvette with a 1 cm path length. The excitation and emission slits were set to 5 and 10 nm, respectively, while the scan speed was maintained at 600 nm.min^−1^. The samples were excited at 280 nm and the emission spectra were recorded in the wavelength range, 300–400 nm. 

## Figures and Tables

**Table 1:** Values of the melting temperatures (Tm), enthalpy variations (ΔH°) and entropy variations (ΔS°) of HSA and AGuIX^®^-HSA complexes with different AGuIX^®^ concentrations (from the SRCD measurements at 192 nm).

Sample	Tm, °C	ΔH°, kJ × mol^−1^	ΔS°, kJ × mol^−1^ × K^−1^
HSA	58.2 ± 0.3	89.3 ± 1.1	0.270 ± 0.003
AGuIX^®^:HSA = 5:1	59.5 ± 0.3	89.5 ± 1.4	0.270 ± 0.003
AGuIX^®^:HSA = 11:1	61.2 ± 0.3	92.0 ± 1.6	0.277 ± 0.003
AGuIX^®^:HSA = 22:1	63.9 ± 0.3	93.6 ± 1.5	0.278 ± 0.003
AGuIX^®^:HSA = 34:1	68.5 ± 0.3	95.2 ± 1.9	0.283 ± 0.003
AGuIX^®^:HSA = 45:1	72.7 ± 0.4	98.7 ± 2.2	0.293 ± 0.003

## Supplementary Materials

Supplementary materials can be found at https://www.mdpi.com/1422-0067/21/13/4673/s1.

## Abbreviations

AGuIX^®^	Activation et Guidage de l’Irradiation X
AgNPsAuNCs	Silver NanoparticlesGold Nanoclusters
AuNPs	Gold Nanoparticles
BSA	Bovine Serum Albumin
CD	Circular Dichroism
DLS	Dynamic Light Scattering
EPR	Enhanced Permeability and Retention
HPLC	High-Performance Liquid Chromatography
HSA	Human Serum Albumin
hTTR	human Transthyretin
ITC	Isothermal Titration Calorimetry
IV	Intravenous
MRI	Magnetic Resonance Imaging
MTD	Maximum Tolerated Doses
NPs	Nanoparticles
PCDDB	Protein Circular Dichroism Data Bank
PET	Positron Emission Tomography
SRCD	Synchrotron Radiation Circular Dichroism
