# Supplementary material for: Human Serum Albumin in the Presence of AGuIX Nanoagents: Structure Stabilisation without Direct Interaction
Source: Int J Mol Sci. 2020 Jun 30;21(13):4673. doi: 10.3390/ijms21134673 (PMC7369717; doi:10.3390/ijms21134673)
Supplement: Supplementary file 1 [file ijms-21-04673-s001.pdf]

International Journal of *Molecular Sciences*

MDPI

Supplementary information

Human Serum Albumin in the presence of AGuIX nanoagents: structure stabilisation without direct interaction

Xiaomin Yang [1], Marta Bolsa-Ferruz [1], Laurent Marichal [2], Erika Porcel [1], Daniela Salado-Leza [1,3], François Lux [4], Olivier Tillement [4], Jean-Philippe Renault [2], Serge Pin [2], Frank Wien [5,*] and Sandrine Lacombe [1,*]

[1] Université Paris-Saclay, CNRS, Institut des Sciences Moléculaires d'Orsay, 91405 Orsay, France;
[2] Université Paris-Saclay, CEA, CNRS, NIMBE, 91191, Gif-sur-Yvette, France;
[3] Cátedras CONACyT, Universidad Autónoma de San Luis Potosí, Facultad de Ciencias Químicas, 78210 San Luis Potosí, S.L.P., Mexico;
[4] Institut Lumière Matière, Université Claude Bernard Lyon 1, CNRS UMR5306, 69622 Villeurbanne, France.
[5] Université Paris-Saclay, Synchrotron Soleil, 91190, Saint-Aubin, France.
* Correspondence: *sandrine.lacombe@universite-paris-saclay.fr (S.L.); *frank.wien@synchrotron-soleil.fr (F.W.); Tel.: +33(1)-6915-8263 (S.L.)

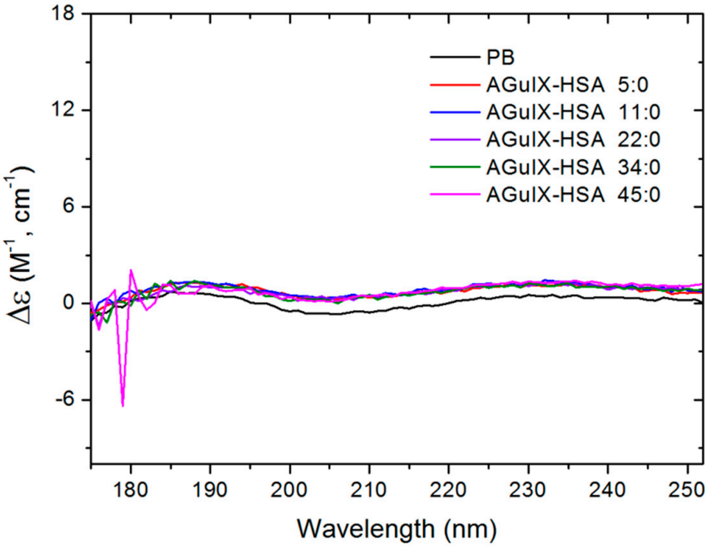

Figure S1. SRCD spectra of control and AGuIX® with increasing concentrations that correspond to ratios from 5:0 to 45:0 in 10 mM phosphate buffer at pH 7.4 and 37°C.

Table S1. Relative content (%) of the secondary structures (alpha helix, turn, random coil) in pure HSA (3100 µg/mL) and in HSA mixed with AGuIX® at various concentrations, pH 7.4 and 37°C (beta sheet 0%).

Sample	% of alpha helix	% of turn	% of random coil
HSA	53±2	14±1	33±2
AGuIX®-HSA 5:1	53±2	14±1	33±2
AGuIX®-HSA 11:1	52±2	15±1	33±2
AGuIX®-HSA 22:1	53±2	15±2	32±2
AGuIX®-HSA 34:1	64±3	16±2	20±2
AGuIX®-HSA 45:1	71±3	7±1	22±2

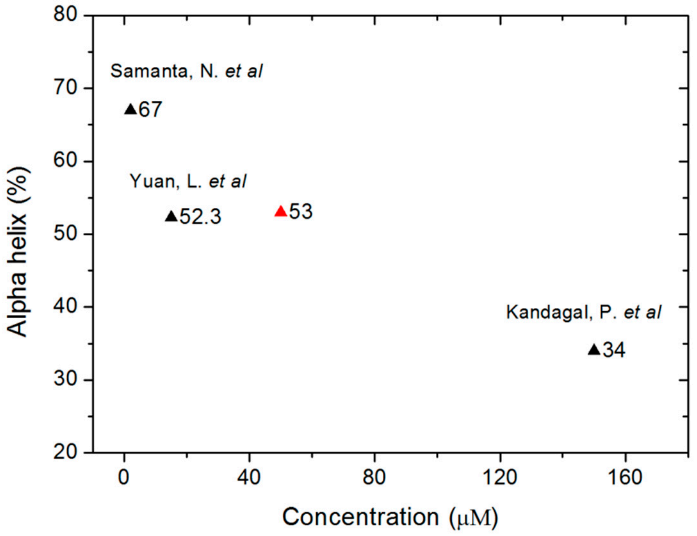

Figure 2. Comparison of the content of alpha helix (%) obtained by our group (red) with the data reported by Yuan, L. et al, Samanta, N. et al and Kandagal, P. et al (black).

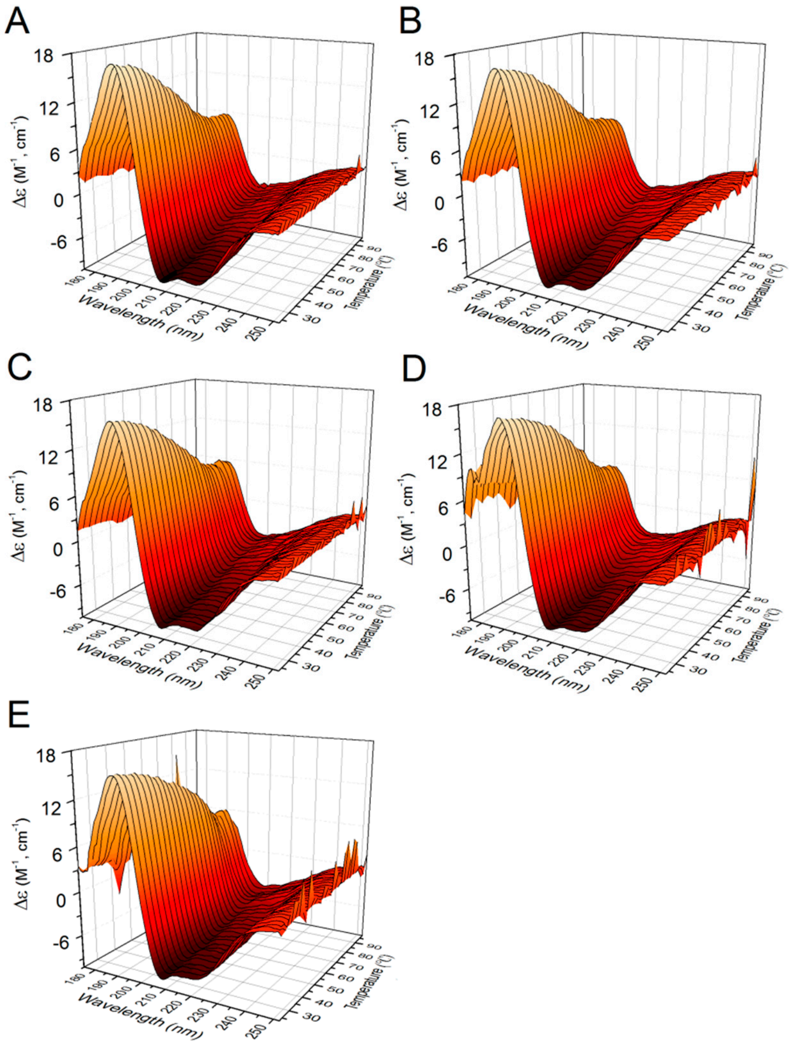

Figure S3. A-E. Temperature dependent SRCD spectra of HSA in 10 mM phosphate buffer at pH 7.4 recorded without (**A**) and in the presence of AGuIX® at the following AGuIX®:HSA ratios: 5:1 (**B**), 11:1 (**C**), 34:1 (**D**) and 45:1 (**D**).

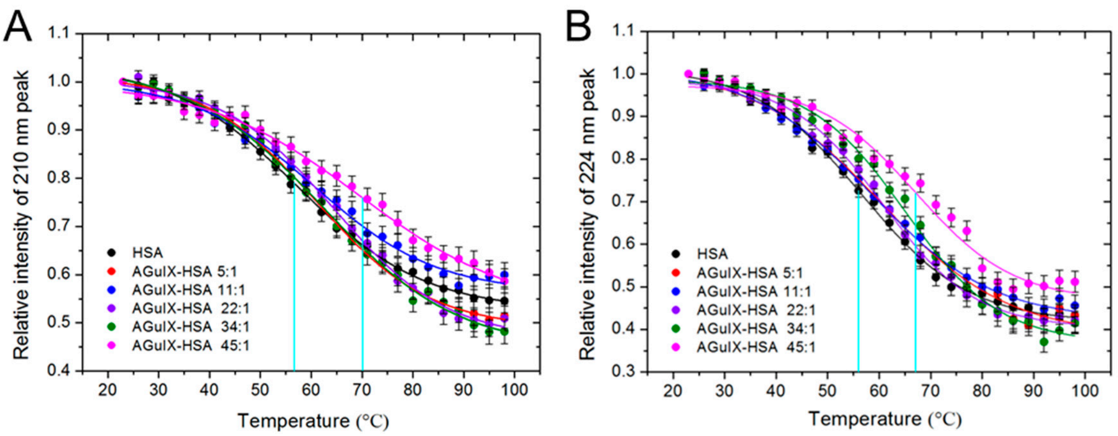

Figure S1. A,B. Relative intensity of the peak at 210 nm and 224 nm for the pure HSA and HSA in the presence of AGuIX® at different ratios. The melting temperatures are determined at half maximum.

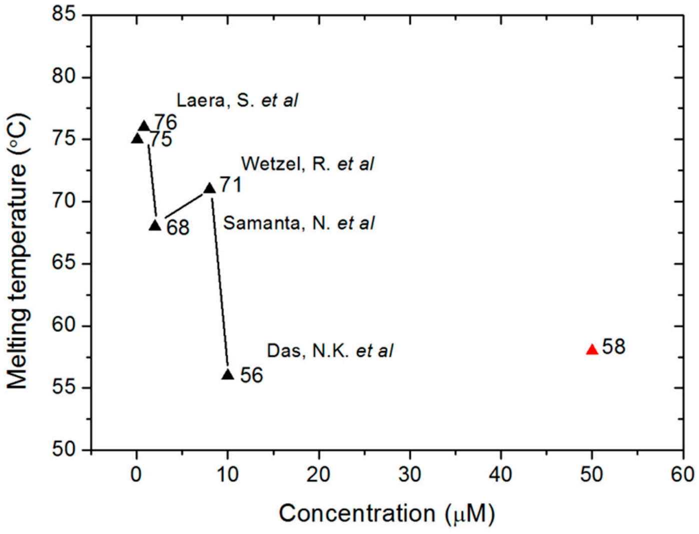

Figure S5. Comparison of melting temperature of pure HSA measured by our group (red) with data reported by different groups (black).

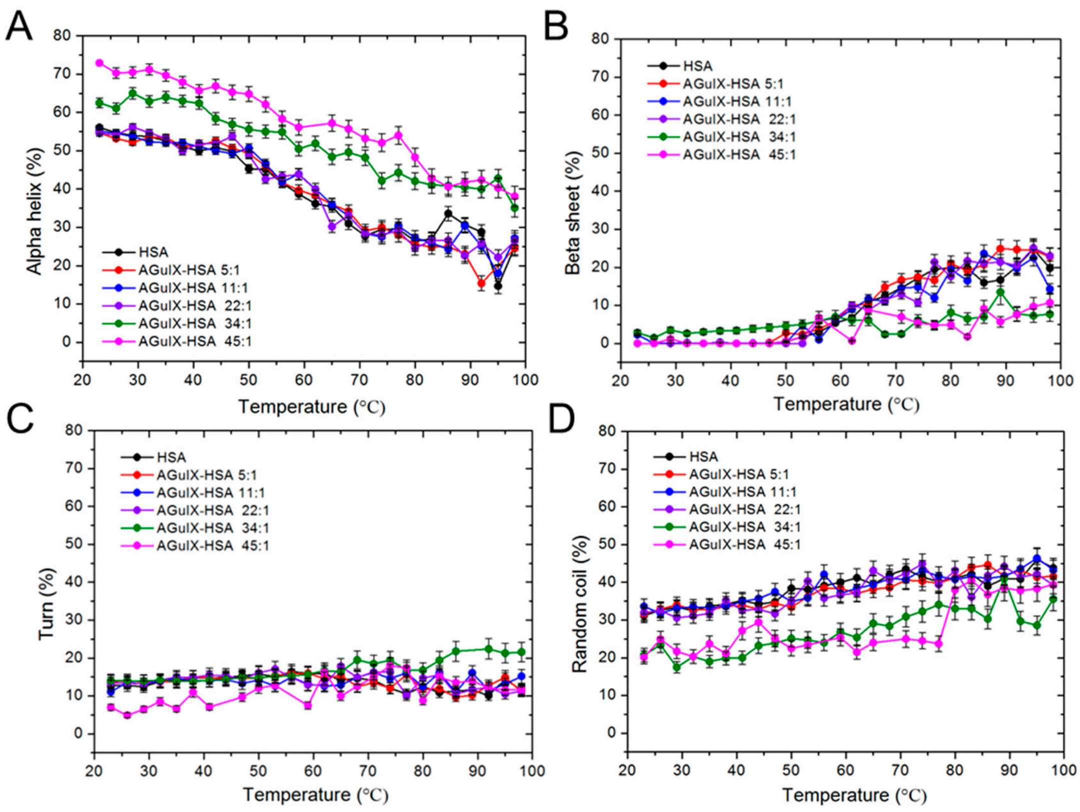

Figure S6. Relative content of alpha-helix (A), beta sheet (B), turn (C), and random coil (D) as function of the temperature for pure HSA and HSA mixed with AGuIX® at different concentrations.

Thermodynamic parameter

The quantitative analysis of protein unfolding is almost exclusively based on the two-state model. It assumes that a protein in solution adopts only two conformational states, the folded (F) and unfolded (U) state. The equilibrium F ↔ U is described with a temperature-dependent equilibrium constant K:

K=[F]/[U]

[F] and [U] are the concentration of the folded and unfolded forms respectively. The fraction folded at any temperature is α.

α =[F]/([F]+[U])

α=K/(1+K)

α = (θt − θU)/(θF − θU)

θt is the observed ellipticity at any temperature, θF is the ellipticity of the fully folded form and θU is the ellipticity of the unfolded form.

The Van't Hoff equation was created to relate equilibrium constant (K) to temperature by substituting the two free energy equations

ΔG°=–RTlnK (at equilibrium, ΔG=0)

ΔG°=ΔH°–TΔS°

and rearranging to generate the Van't Hoff equation

$$\ln K = \frac{-\Delta H°}{RT} + \frac{\Delta S°}{R}$$

Where the natural logarithm (ln) of the folding constant, K is plotted as a function of 1/T, T is the absolute temperature (Kelvin) and R is the universal gas constant=8.314J·mol-1·K-1. From the graph analysis, we obtain ΔH° (from the slope of the plot -ΔH°/R) and ΔS°(from the intercept of the plot ΔS°/R). The values are reported in the table S2.

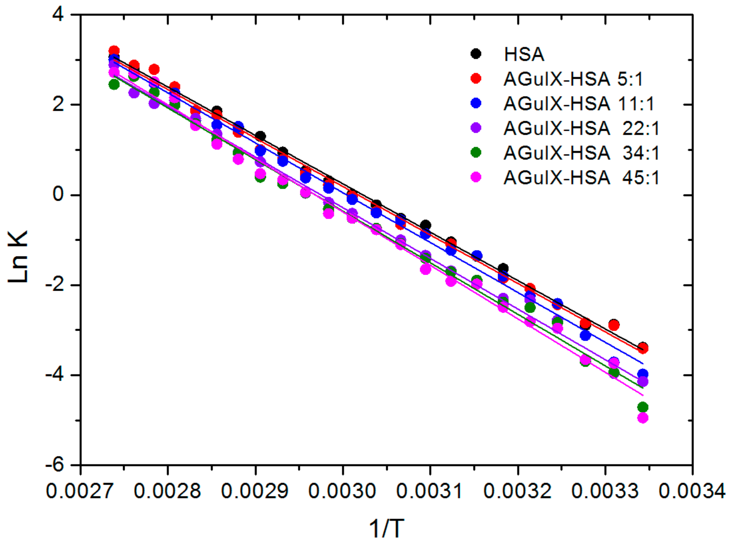

Figure S2. Van't Hoff plot for the calculation of thermodynamic parameters of the pure HSA and in the presence of AGuIX® at ratios from 5:1 to 45:1 (at 192 nm).

Table S1. Values of the slope, intercept and R^2 of the Van't Hoff plot of HSA and AGuIX®-HSA with different AGuIX® concentrations (from the SRCD measurements at 192 nm).

Sample	Slope	Intercept	R^2
HSA	-10742.1±130.8	32.475±0.399	0.997
AGuIX®-HSA 5:1	-10759.8±167.7	32.476±0.407	0.995
AGuIX®-HSA 11:1	-11071.3±202.6	33.265±0.406	0.994
AGuIX®-HSA 22:1	-11253.0±183.0	33.477±0.414	0.994
AGuIX®-HSA 34:1	-11455.6±224.0	34.008±0.418	0.993
AGuIX®-HSA 45:1	-11875.1±254.9	35.251±0.420	0.991

UV-vis absorption spectra

A CARY 300 spectrophotometer (Int. Agilent) was used to scan the UV-visible spectra. For the inner filter effect correction of the fluorescence quenching data, the UV-visible absorption spectra of HSA (4.7 μM) in the absence and presence of AGuIX® at different ratios (5:1 to 45:1) in a total volume of 1 mL were recorded at the 250–400 nm wavelength range.

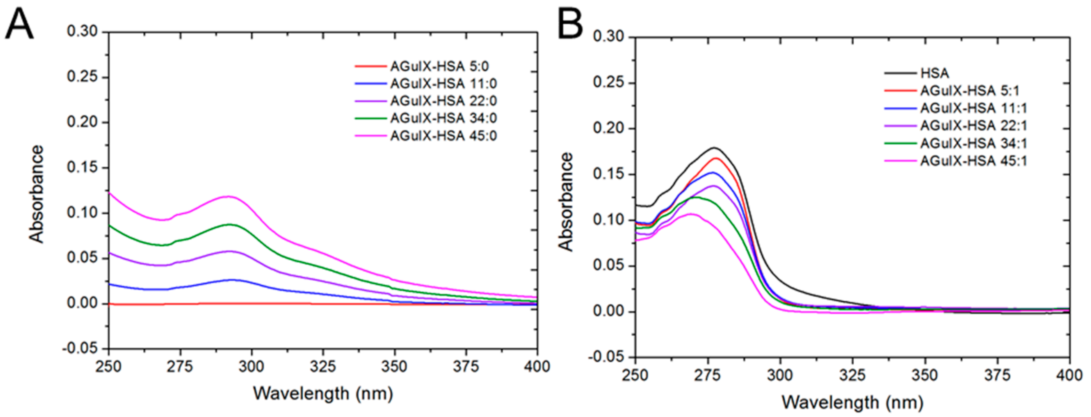

Figure S3. (A) UV-vis absorption spectra of free AGuIX® at different concentrations from 5:0 to 45:0 in 67mM phosphate buffer, pH 7.4. (B) UV-vis absorption spectra of HSA without and with increasing AGuIX® concentrations (AGuIX®:HSA ratios from 5:1 to 45:1) in 67mM phosphate buffer, pH 7.4. The spectra were obtained after subtracting the absorption spectra of AGuIX® from the respective absorption spectra of AGuIX®-HSA samples.

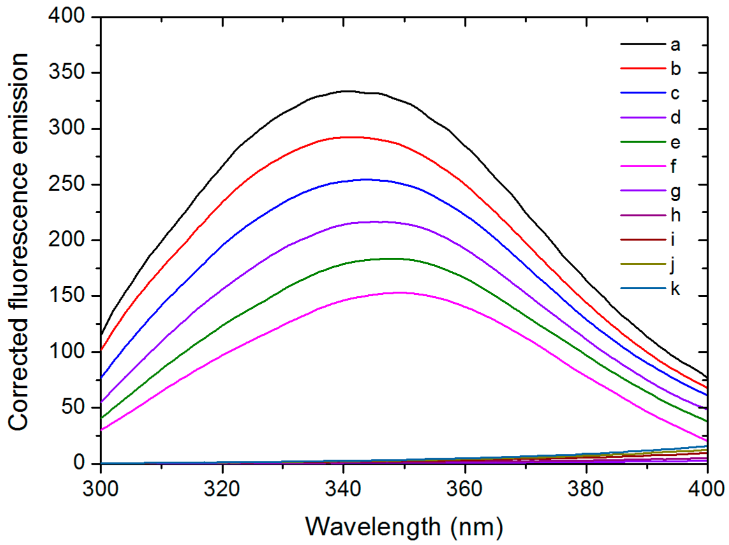

Figure. S4. Corrected fluorescence emission spectra of HSA without (a) and with increasing AGuIX® concentrations from 5:1 to 45:1 (b-f), AGuIX® alone at corresponding concentration (g-k) in 67mM phosphate buffer, pH 7.4 at 37°C upon excitation at 280 nm.

Table S3. The Stern-Volmer constant (Ksv, M-1) and the quenching rate constant (kq in M-1.s-1) characteristics of the AGuIX®-HSA interactions, at 25°C and 37°C, pH 7.4.

T/°C	Ksv, M^{-1}	kq, $M^{-1} \cdot s^{-1}$
25	5.2×10^{3}	8.15×10^{11}
37	5.9×10^{3}	9.25×10^{11}
